# Temporal effects on death by suicide: empirical evidence and possible molecular correlates

**DOI:** 10.1007/s44192-023-00035-4

**Published:** 2023-04-03

**Authors:** R. Bhagar, H. Le-Niculescu, K. Roseberry, K. Kosary, C. Daly, A. Ballew, M. Yard, G. E. Sandusky, A. B. Niculescu

**Affiliations:** 1 Department of Psychiatry, Indiana University School of Medicine, Neuroscience Research Building 200B, 320 W. 15thStreet, Indianapolis, IN 46202 USA; 2 Marion County Coroner’s Office, Indianapolis, IN USA; 3 INBRAIN, Indiana University School of Medicine, Indianapolis, IN USA; 4 Indianapolis VA Medical Center, Indianapolis, USA

**Keywords:** Full moon, Suicide, Biomarkers, Age differences

## Abstract

Popular culture and medical lore have long postulated a connection between full moon and exacerbations of psychiatric disorders. We wanted to empirically analyze the hypothesis that suicides are increased during the period around full moons. We analyzed pre-COVID suicides from the Marion County Coroner’s Office (n = 776), and show that deaths by suicide are significantly increased during the week of the full moon (p = 0.037), with older individuals (age ≥ 55) showing a stronger effect (p = 0.019). We also examined in our dataset which hour of the day (3–4 pm, p = 0.035), and which month of the year (September, p = 0.09) show the most deaths by suicide. We had blood samples on a subset of the subjects (n = 45), which enabled us to look at possible molecular mechanisms. We tested a list of top blood biomarkers for suicidality (n = 154) from previous studies of ours ^7^, to assess which of them are predictive. The biomarkers for suicidality that are predictive of death by suicide during full moon, peak hour of day, and peak month of year, respectively, compared to outside of those periods, appear to be enriched in circadian clock genes. For full moon it is AHCYL2, ACSM3, AK2, and RBM3. For peak hour it is GSK3B, AK2, and PRKCB. For peak month it is TBL1XR1 and PRKCI. Half of these genes are modulated in expression by lithium and by valproate in opposite direction to suicidality, and all of them are modulated by depression and alcohol in the same direction as suicidality. These data suggest that there are temporal effects on suicidality, possibly mediated by biological clocks, pointing to changes in ambient light (timing and intensity) as a therapeutically addressable target to decrease suicidality, that can be coupled with psychiatric pharmacological and addiction treatment preventive interventions.

**Supplementary Information:**

The online version contains supplementary material available at 10.1007/s44192-023-00035-4.

## Introduction

“It is the very error of the moon:

She comes more nearer earth than she was wont,

And makes men mad.”

-. William Shakespeare

Full moon periods have historically and anecdotally been associated with mental illness and its exacerbations. Previous evidence has been mixed. A review study looking at a broad range of psychiatric behaviors (psychosis related admissions, suicide attempts, ER visits, and other indicators of psychiatric illness), found that these “abnormal behaviors” occurred at a greater frequency during the New Moon and Full Moon periods [1]. There has also been work that has shown the impact of the lunar phases on those diagnosed with bipolar disorder [2–5]. For example, subjects with rapid-cycling bipolar seemed to entrain on lunar cycles (noting potential for both the moon’s luminance and gravitational tidal cycles having impacts) [2, 5]. Clinically, it is known that those suffering from bipolar disorder typically struggle in regards to both seasonal and circadian rhythms, as well as the fact that age of onset of bipolar disorder varies based on distance from the equator [4]. In regards to suicide it has been shown that the variability and amount of total sunlight throughout the year can impact suicide rate in those with bipolar disorder, and those who experience large variations throughout the year in regards to sun exposure are at elevated risk for suicide [3]. This highlights that in addition to the impact of lunar cycles as noted above, seasonality, geography, and overall sun exposure seem to have relationship with psychiatric diseases. Additionally, although a Finnish study in 2021 showed lack of evidence for a broad relationship between suicidality and moon phase, there was shown to be a statistically significant correlation between suicide and the full moon, and further analysis revealed a peak of incidence of suicide during full moons during the winter for pre-menopausal women [6]. This highlights the fact that in conjunction with consideration of lunar phase, seasonal effect should be considered as well, as was mentioned earlier.

We wanted to examine empirically if the above effects of full moon on suicides are true. We also examined empirically other potential temporal effects, such as peak time of day and peak month of year. For that, we studied a pre-COVID cohort of suicide completers from an urban coroner’s office. We also conducted molecular blood biomarkers analyses on a subset of the subjects on which we had blood samples.

## Materials and methods

### Cohort

Marion County Coroner’s Office cases of deaths by suicide from January 1, 2012 to December 17, 2016 were collected from coroners log books and files. As these were post-mortem cases, the need for an active IRB protocol and informed consent for these cases was waived by the Indiana University IRB. The log books contained the date of death (or date found), case number, preliminary manner of death, name, age, sex, and race along with the name of the responding deputy coroner. If there was a question regarding the manner of death, the case numbers were marked and checked by a Deputy Coroner using their database. All data was entered into a spreadsheet in a de-identified fashion. In all, there were 210 completed suicides over a period of 626 days within the week of the full moon and 566 suicides over a period of 2006 days outside the week of the full moon. Completed suicides involving individuals aged 30 years and younger (n = 208) and 55 years and older (n = 232) were also analyzed separately (Table S1).

A subset of cases had blood samples collected as part of our INBRAIN initiative (Indiana Center for Biomarker Research in Neuropsychiatry) (Supplementary Table S2). We required a last observed alive postmortem interval of 24 h or less, and the cases selected had completed suicide by means other than overdose, which could affect gene expression. The 45 total samples consisted of 38 male and 7 female violent suicide completers (Table S1). 31 participants completed suicide by gunshot to head or chest, 12 by asphyxiation, 1 by slit wrist, and 1 by electrocution. Next of kin signed informed consent at the coroner’s office for donation of blood for research. We collected whole blood (10 ml) in two RNA-stabilizing PAXgene tubes, labeled with an anonymized ID number, and stored at −80 °C in a locked freezer until the time of future processing. Whole-blood RNA was extracted for microarray gene expression studies from the PAXgene tubes, as previously described [7].

### Clock gene analysis

We annotated the suicide biomarker genes for involvement in the circadian clock. We compiled a database of genes associated with circadian function, by using a combination of review papers [8, 9] and searches of existing databases CircaDB (http://circadb.hogeneschlab.org), GeneCards (http://www.genecards.org), and GenAtlas (http://genatlas.medecine.univ-paris5.fr). Using the data we compiled from these sources we identified a total of 1468 genes that show circadian functioning. Using an estimate of about 21,000 genes in the human genome, that gives about 7% of genes having some circadian pattern. We further classified genes into “core” clock genes, i.e. those genes that are the main engine driving circadian function (n = 18), “immediate” clock genes, i.e. the genes that directly input or output to the core clock (n = 331), and “distant” clock genes, i.e. genes that directly input or output to the immediate clock genes (n = 1,119). Out of our 154 top biomarker genes [7], 18 had circadian evidence (11.7%) suggesting a 1.7 fold enrichment for circadian genes.

### Literature databases search

Databases. We have established in our laboratory (Laboratory of Neurophenomics, www.neurophenomics.info) manually curated databases of the human gene expression/protein expression studies (postmortem brain, peripheral tissue/fluids: CSF, blood and cell cultures), human genetic studies (association, copy number variations and linkage), and animal model gene expression and genetic studies, published to date on psychiatric disorders. Only findings deemed significant in the primary publication, by the study authors, using their particular experimental design and thresholds, are included in our databases. Our databases include only primary literature data and do not include review papers or other secondary data integration analyses to avoid redundancy and circularity. These large and constantly updated databases have been used in our CFG cross validation and prioritization platform. For this study, data from papers on suicide were used for the primary analyses (Table 1), and on other disorders for co-morbidity analyses (Table S3).

**Table 1:** Genomics

Time period	% Clock genes out of the suicidality biomarkers that predict death during peak time periods	Clock gene (AUC/p-value)	Clock gene category	ANOVA p-value (no SI vs. high SI vs. Suicide completers) [7]	Other human evidence for suicidality	Drugs that modulate the clock gene in the opposite direction to suicide
Full moon	All 66.6% (2/3)	(D) AHCYL2 0.733/0.016	Distant output	Stepwise 6.28E-05 Bonferroni	(I) NAC suicide [15]	
		(D) RBM3 0.722/0.021	Distant output	Stepwise 1.73E-05 Bonferroni	(D)Hippocampus suicide [16] (D) DLPFC females suicide [17]	(I) Lymphoblastoid cells Lithium [18] (I) Dentate gyrus Fluoxetine [19] (I) PFC Valproate [20] (I) Hippocampus (CA1) Gamma frequency [21] (I) Lymphocytes (females) Omega-3 fatty acids [22]
	≥ 55 yo 15.4% (2/13)	(D) RBM3 0.939/0.012	Distant output	Stepwise 1.73E-05 Bonferroni	(D) Hippocampus suicide [16] (D) DLPFC females suicide [17]	(I) lymphoblastoid Lithium [18] (I) Dentate gyrus Fluoxetine [19] (I) PFC Valproate [20] (I) Hippocampus (CA1) Gamma frequency [21] (I) Lymphocytes (females) Omega-3 fatty acids [22]
		(D) AHCYL2 0.848/0.037	Distant output	Stepwise 6.28E-05 Bonferroni	(I) NAC suicide [15]	
	≤ 30 yo 25% (3/12)	(D) RBM3 0.846/0.035	Distant output	Stepwise 1.73E-05 Bonferroni	(D) Hippocampus suicide [16] (D) DLPFC females suicide [17]	(I) lymphoblastoid Lithium [18] (I) Dentate gyrus Fluoxetine [19] (I) PFC Valproate [20] (I) Hippocampus (CA1) Gamma frequency [21] (I) Lymphocytes (females) Omega-3 fatty acids [22]
		(D) AK2 0.821/0.046	Distant output	Stepwise 3.19E-06 Bonferroni	Suicide Genetic Association [23]	(I) Blood Escitalopram (SSRI) [24]
		(D) ACSM3 0.820/0.046	Distant output	Stepwise 9.67E-06 Bonferroni	(I) PFC suicide [25]	(I) Embryonic stem cell Carbamazepine [26] (I) Hippocampus Valproic acid [27]
Peak hour	All 100% (1/1)	(D) GSK3B 0.706/0.05	Immediate output	Stepwise 2.19E-36 Bonferroni	(D) PFC suicide [28] (D) PFC suicide [29] (D) Locus Coeruleus Suicide [30] (D) PFC suicide [31] Suicide Genetic Association [32]	(I) Lithium [33] (I) Blood mononuclear cells Lithium [34] (I) Lymphoblastoid cell lines (LCLs) Lithium [35] (I) Corpus Collosum Lithium [36] (I) Hippocampus Baicalin [37] (I) Platelets mood stabilizers [38] (I) Astrocytes Carbamazepine, Lithium, Valproic Acid [39] (I) Blood sleep deprivation [40] (I) Cell culture Paliperidone [41] (I) AMY Valproate [20] (I) Frontal cortex Haloperidol, Lithium [42] (I) PFC Paliperidone [43] (I) Striatum Pregnenolone sulfate [44] (I) Cortex Fluoxetine [45]
	≥ 55 25% (2/8)	(D) AK2 0.955/0.024	Distant output	Stepwise 1.15E-07 Bonferroni	Suicide Genetic Association [23]	(I) Blood Escitalopram (SSRI) [24]
		(D) PRKCB 0.955/0.024	Distant output	Stepwise 2.40E-13 Bonferroni	(D) DLPFC suicide [46] (D) DLPFC suicide [47] (I) DLPFC suicide [17] (D) PFC, hippocampus suicide [48] (I)Blood suicide in veterans [49]	(I) Embryonic stem cell Valproate [26] (I) Cerebral Cortex (right) Lithium [50] (I) AMY Lithium [51] (I) Fetal Brain Acetaminophen [52]
	≤ 30 0% (0/0)	No significant predictors				
Peak month	All (September) 0% (0/11)	No clock genes				
	≥ 55 (July) –% (-/-)	No blood samples in July				
	≤ 30 (November) 18.18% (2/11)	(D) TBL1XR1 0.923/0.028	Distant output	Stepwise 2.34E-08 Bonferroni	Suicide Genetic Association [53]	(I) VT Clozapine [54]
		(D) PRKCI 0.893/0.040	Distant output	Stepwise 2.71E-05 Bonferroni	(I) PFC (BA 46) suicide [55] (I) DLPFC females completers [17]	(I) Corpus Callosum Lithium [36]

Clock Genes Enrichment in Suicidality Biomarkers. Baseline, 11.7% (18/154) % Clock Genes out of All Suicidality Biomarkers [7]. That % appears higher in the full moon, peak hour, and peak months periods. (I)-Increased in Expression. (D)- Decreased in Expression. PFC- Pre-frontal Cortex. AMY-amygdala

### Predicting temporal windows (full mood, peak hour of day, peak month of year)

The cohort (n = 45) for predicting temporal windows had whole-genome Affymetrix gene expression data [7] that was RMA normalized by gender. Predictions were performed using R-studio. A list of 154 top biomarkers for suicidality [7] was used. Receiver-operating characteristic (ROC) analyses were carried out between temporal window and outside of the temporal window. We used the pROC function of the R studio.

## Results

### Phenomenology (Fig. 1)

**Fig. 1:**
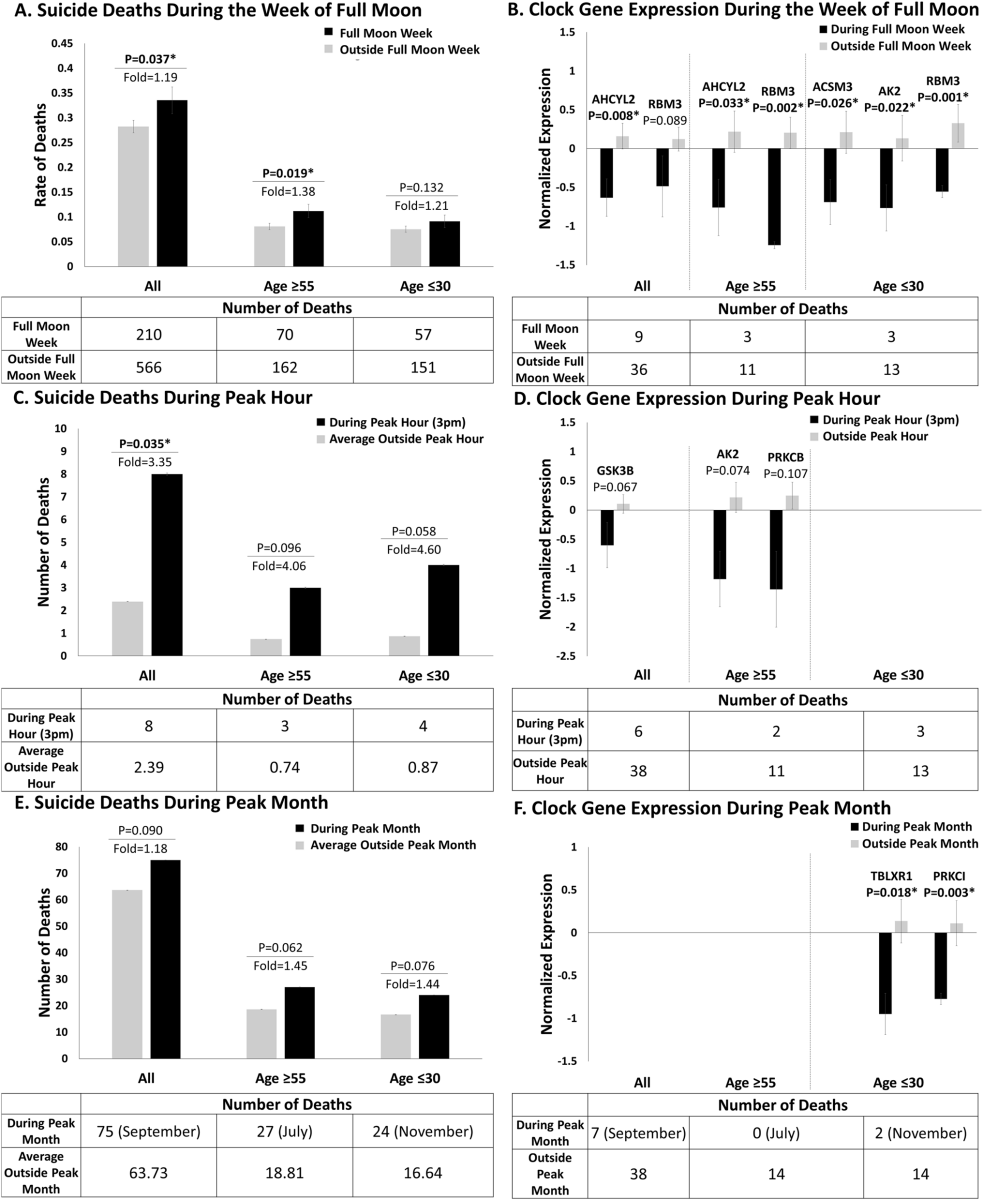
Phenomics and Genomics. **A**,**C**, **E**. Phenomenological data during the peak time periods. T-tests and standard error of mean (SEM). **B**, **D**, **F**. Gene expression data during the peak time periods. Data in normalized (Z-scored) by gender. The genes whose expression is depicted are the clock genes from Table 1. Clock genes are enriched above baseline among the suicidality biomarkers that are significantly predictive of the peak time periods

Suicides deaths were increased during the week of the full moon (p = 0. 037), in particular in over 55 years old (p = 0.019). There was no statistically significant increase in under 30 years old (p = 0.132).

The peak hour of the day for suicides was 3 to 4 pm (p = 0.035). The peak month was September in all, with July being the peak month in over 55 years old and November being the peak month in under 30 years old. None of these reached statistical significance.

### Genomics (Fig. 1 and Table 1)

Clock genes were enriched among suicidality biomarkers that were predictors of the temporal windows (Table 1), compared to the baselines of 7% of clock genes in the genome, and 11.7% of clock genes among the top suicidality biomarkers [7].

### Co-morbidity, treatments, and biological roles (Table 2 and Table S4)

**Table 2:** Clinical insights

A	
Co-morbidity	Percentile match (%)
Depression	100
Alcohol	100
Stress	87.5
Aging	75
Dementia	75
BP	62.5
SZ	62.5
PTSD	37.5
Suicide	37.5
Anxiety	25
Methamphetamine	25
Autism/ASD	12.5
Hallucinogens	12.5
Neurological (Huntington,ALS)	12.5
Pain	12.5
Parkinson	12.5
Psychosis	12.5
Substance abuse	12.5

B	
Treatment	Percentile match (%)
Lithium	50
Valproic acid	50
Carbamazepine	25
Fluoxetine	25
Baicalin	12.50
Clozapine	12.50
Escitalopram	12.50
Gamma frequency	12.50
Omega-3 fatty acids	12.50
Paliperidone	12.50
Paracetamol (acetaminophen)	12.50
Pregnenolone sulfate	12.50
Sleep deprivation	12.50

A. Co-morbidity % of genes in Table 1 that are changed in expression in same direction as in suicide B. Therapeutics. % of genes in Table 1 that are modulated in expression by existing treatments in direction opposite to the one in suicide

Depression and alcoholism were the top co-morbid disorders, lithium and valproate the top treatments, and Wnt and Errb signaling were the top biological pathways.

## Discussion

An increase in suicides during the full moon could be due to the moonlight affecting vulnerable individuals at a time when there should be dark. Consistent with that, circadian clock genes are enriched for predicting this temporal interval. Moreover, the age differential may be consistent as well. For this data, primarily from prior to 2016, the nighttime exposure to light is arguably lower in those over 55 years old, as they were using less cell phones and going to sleep earlier than those under 30 years old. As such, the abnormal light from a full moon may be perceived by them more strongly, whereas it would be drowned by other sources of ambient light in the younger individuals.

The peak of suicides at 3–4 pm is intriguing, and again could have psycho-social and biological reasons. The psycho-social reasons may include day of event stressors such as work events triggering people to leave work early and complete suicides, at a time when they are alone at home. The biological reasons may be circadian clock related, with a decrease in light starting to occur at that time of day and a lower expression of circadian clock genes, as well as cortisol. Indeed, a decrease in gene expression of the clock genes GSK3B, AK2 and PRKCB was predictive in our data (Table 1).

The peak for suicides during September, incidentally, coinciding with Suicide Prevention Month, may be due as well to psycho-social and biological reasons. The psycho-social reasons may have to do with summer vacations being over, and work and school restarting, which may place stress on vulnerable individuals. The biological reasons may be circadian clock related seasonal affective disorder effects, with a decrease in daylight occurring at that time of year. Psycho-socially, the peaks in July for over 55 years old and in November for under 30 years old may be due to US holidays without or with relatives differentially affecting these age groups. Biologically, the increase daylight in July may give older people the energy to do something, and the decreased daylight in November may make younger people sad (they have in general sufficient energy at that age to do something regardless of time of year).

Previous work by us and others has shown that circadian clock abnormalities are related to mood disorders [9–13], and sleep abnormalities have been implicated in suicide [14]. The enrichment in, and putative involvement of, circadian clock genes in death by suicide in the peak temporal windows provides insights and opens the door to therapeutic interventions, whether chronobiological or pharmacological. The co-morbidity with depression and alcoholism may help in building a risk profile, and it is noted that lithium and valproate may be suitable drugs to prevent suicides during peak temporal risk times and beyond (Table 2).

In conclusion, our work is supportive of the full moon, fall season, and late afternoon being temporal windows of increased risk for suicide, where vigilance and preventive measures are warranted, particularly in individuals who suffer from depression and/or alcohol use disorders.

## Supplementary Information

Below is the link to the electronic supplementary material.

Supplementary file1 (DOCX 190 KB)

## Data Availability

The datasets generated during and/or analyzed during the current study are available from the corresponding author on reasonable request.
