# Supplementary material for: Temporal effects on death by suicide: empirical evidence and possible molecular correlates
Source: Discov Ment Health. 2023 Apr 3;3(1):10. doi: 10.1007/s44192-023-00035-4 (PMC10501025; doi:10.1007/s44192-023-00035-4)
Supplement: Supplementary file 1 — Supplementary file1 (DOCX 190 KB) [file 44192_2023_35_MOESM1_ESM.docx]

**Table S1. Demographics**

|  | **Number of Subjects** | **Gender** | **Age Mean**  **(SD)** | **Ethnicity** |
| --- | --- | --- | --- | --- |
| **Phenomics** | | | | |
| **All** | 776 | Male=621  Female=155 | 44.50  (18.07) | EA=645  AA=98  Hispanic=19  Asian=13  NA=1 |
| **Inside Week of Full Moon** | 210 | Male=164  Female=46 | 45.60  (18.47) | EA=180  AA=23  Hispanic=5  Asian=2 |
| **Outside Week of Full Moon** | 566 | Male=457  Female=109 | 44.09  (17.92) | EA=465  AA=75  Hispanic=14  Asian=11  NA=1 |
| **During Peak Hour (3-4PM)** | 8 | Male=6  Female=2 | 40.50  (16.80) | EA=7  AA=1 |
| **Outside Peak Hour** | 55 | Male=49  Female=6 | 39.83  (16.89) | EA=46  AA=6  Asian=1  Hispanic=1 |
| **During Peak Month (September)** | 75 | Male=60  Female=15 | 44.65  (19.51) | EA=69  AA=4  Hispanic=1  Asian=1 |
| **Outside Peak Month (September)** | 701 | Male=688  Female=13 | 44.48  (17.93) | EA=694  AA=5  Hispanic=1  Asian=1 |
| **Genomics** | | | | |
| **All** | 45 | Male=38  Female=7 | 40.69  (16.93) | EA=37  AA=7  Hispanic=1 |
| **Inside Week of Full Moon** | 9 | Male=8  Female=1 | 43.11  (15.82) | EA=9 |
| **Outside Week of Full Moon** | 36 | Male=30  Female=6 | 40.08  (17.36) | EA=28  AA=7  Hispanic=1 |
| **During Peak Hour (3-4 PM)** | 6 | Male=5  Female=1 | 39.50  (16.37) | EA=5  AA=1 |
| **Outside Peak Hour** | 38 | Male=32  Female=6 | 40.87  (17.21) | EA=32  AA=6 |
| **During Peak Month (September)** | 7 | Male=7 | 39.29  (16.95) | EA=7 |
| **Outside Peak Month** | 38 | Male=31  Female=7 | 40.95  (17.14) | EA=30  AA=7  Hispanic=1 |

**Table S2: Suicide completers for which we had blood samples (n=45).**

|  | | | | | | | |
| --- | --- | --- | --- | --- | --- | --- | --- |
| Subject ID | Psych Dx | Date of Death | Age | Gender | Ethnicity | Cause of Death | Toxicology Report |
| INBRAIN09 | Bipolar/schizophrenia | 5/27/2011 | 59 | M | Caucasian | Hanging |  |
| INBRAIN011 | Depression/ADHD | 9/10/2011 | 26 | M | Caucasian | GSW to chest | ALPRAZOLAM 3.2 NG/ML TRAMADOL 331 NG/ML NORTRAMADOL 179 NG/ML BUPROPION 136 NG/ML CITALOPRAM/ESCITALOPRAM 229 NG/ML CAFFEINE POSITIVE COTININE POSITIVE |
| INBRAIN012 | Depression, untreated | 9/20/2011 | 39 | M | Caucasian | GSW to head | Negative |
| INBRAIN013 | Depression | 1/4/2012 | 68 | M | African American | GSW to mouth | CAFFEINE POSITIVE |
| INBRAIN014 | None | 2/9/2012 | 27 | M | Caucasian | Hanging | ETHANOL 0.15 % (W/V) CAFFEINE |
| INBRAIN015 | None | 3/12/2012 | 40 | M | Caucasian | Hanging | ETHANOL 0.119 % (W/V) CAFFEINE THC testing cancelled due to interference? |
| INBRAIN016 | Anxiety/TBI | 4/15/2012 | 68 | M | Caucasian | GSW to right ear area | Diazepam 155 Nordiazepam 61.9 Alprazolam 6.8 Atenolol Warfarin Caffeine |
| INBRAIN017 | Depression | 4/28/2012 | 56 | M | Caucasian | GSW to the chest area | Clonazepam 6 6 7-Aminoclonazepam 73.7 Glucose positive urine THC 2.0 THC-COOH 10.5 Ethanol 0.130 Fluoxetine 636 Norfluoxetine 359 Venlafaxine 1641 Norvenlafaxine 136 Caffeine |
| INBRAIN018 | None | 5/15/2012 | 65 | M | Caucasian | A deep cutting injury to his left wrist area | ETHANOL 0.057 %(W/V) AMIODARONE CAFFEINE COTININE |
| INBRAIN019 | Depression | 12/13/2012 | 55 | M | Caucasian | GSW to head & chest | Alprazolam 169 Caffeine |
| INBRAIN020 | Depression | 12/18/2012 | 55 | F | Caucasian | GSW to the Chest area | clonazepam 6.7, 7=aminoclonazepam 32.9, duloxetine 68.7, trazodone 0.21 |
| INBRAIN021 | Depression | 1/10/2013 | 23 | M | African American | hanging | THC 8.9 THC 60.2 Isopropanol 0.042 |
| INBRAIN022 | Depression | 1/30/2013 | 38 | M | Hispanic | GSW to head | Ethanol 0.185 Caffeine |
| INBRAIN023 | None | 2/11/2013 | 18 | M | Caucasian | suicide by hanging | CAFFEINE POSITIVE |
| INBRAIN024 | None | 2/17/2013 | 23 | M | Caucasian | suicide by hanging | CAFFEINE POSITIVE |
| INBRAIN025 | None | 2/20/2013 | 31 | M | African American | Poss murder suicide: GSW to the head | THC 1.2 THC-COOH 12.0 Caffeine |
| INBRAIN026 | NP | 2/28/2013 | 57 | F | Caucasian | GSW to head | CAFFEINE POSITIVE |
| INBRAIN028 | Alcoholism | 4/27/2013 | 67 | M | Caucasian | Single GSW to chest | Ethanol 0.354 ANTIHISTAMINES Diphenhydramine 178 Amlodipine 19.9 Caffeine |
| INBRAIN029 | PTSD | 5/3/2013 | 36 | F | Caucasian | Suicide by asphyixiation (Duct tape) | NA |
| INBRAIN030 | None | 5/4/2013 | 22 | M | African American | GSW to head (Suicide) | CAFFEINE POSITIVE |
| INBRAIN032 | Bipolar | 5/10/2013 | 44 | F | Caucasian | GSW to head | CAFFEINE POSITIVE |
| INBRAIN033 | Depression | 5/19/2013 | 26 | M | Caucasian | GSW to his left chest | Ethanol 0.128 Citalopram 294 Caffeine |
| INBRAIN034 | Depression | 6/15/2013 | 50 | F | Caucasian | GSW to his left chest | Oxazepam 54.5, Temazepam 395, Gabapentin 1, Zolpidem 571, Temazepam >2500, Oxazepam>2500, Hydrocodone 88, Hydromorphine 161 |
| INBRAIN035 | Depression | 9/20/2013 | 58 | M | Caucasian | Electrocution | Venlafaxine 231 Norvenlaxfaxine 452 Amlodipine 45.3 Caffeine |
| INBRAIN036 | None | 9/23/2013 | 59 | M | Caucasian | GSW to chest | Negative |
| INBRAIN039 | None | 10/16/2013 | 53 | M | Caucasian | Hanging | Ethanol 0.158 Caffeine |
| INBRAIN040 | Depression | 10/31/2013 | 36 | M | Caucasian | GSW- head | Ibuprofen 8.2 Caffeine |
| INBRAIN041 | Depression | 11/25/2013 | 76 | M | Caucasian | GSW- head | CAFFEINE POSITIVE |
| INBRAIN044 | None | 3/30/2014 | 23 | M | Caucasian | Hanging | THC 10.3 THC-COOH 143 |
| INBRAIN048 | Schizophrenia | 5/17/2014 | 26 | M | Caucasian | GSW- head | CARBOXY THC 78 ng/ml CAFFEINE |
| INBRAIN050 | Depression | 8/24/2014 | 19 | F | African American | GSW- Head |  |
| INBRAIN051 | None | 8/26/2014 | 17 | M | African American | Suicide hanging | Oxazepam, Temazepam, Cannabinoids, THC, THC-COOH, Analgesics, Antidepressants, Bupropion, Stimulants (Caffeine), Benzodiazepines, Temazepam, Nordiazepam, Oxazepam |
| INBRAIN052 | None | 9/12/2014 | 16 | M | Caucasian | GSW - Head | None |
| INBRAIN053 | Depression | 9/16/2014 | 50 | M | Caucasian | GSW- Neck | pending |
| INBRAIN054 | Depression | 9/20/2014 | 27 | M | Caucasian | GSW - Shotgun to left chest | Opiates (Hydrocodone, Hydromorphone), Alcohol(Ethanol), Analgesics (Acetaminophen, Cyclobenzaprine), Stimulants (Caffeine), |
| INBRAIN055 | Depression | 11/20/2014 | 18 | M | Caucasian | GSW - Head | THC-COOH 4.3 ng/mL; Caffeine (Presumptively positive, result not confirmed by alternate method); Carboxy THC 69 ng/mL |
| INBRAIN056 | None | 12/3/2014 | 37 | M | Caucasian | Hanging | Negative |
| INBRAIN058 | None | 1/30/2015 | 55 | M | Caucasian | Single GSW to head |  |
| INBRAIN059 | Depression | 3/26/2015 | 43 | M | African American | GSW - Head | Antidepressants (Citalopram/Escitalopram), Stimulants (Caffeine) |
| INBRAIN060 | None | 4/16/2015 | 28 | M | Caucasian | GSW - Head | Alcohol(Ethanol) |
| INBRAIN061 | None | 5/5/2015 | 37 | M | Caucasian | GSW - Head | Opiates (Morphine, Monoacetylmorphine, Codeine), Stimulants (Caffeine) |
| INBRAIN062 | None | 5/11/2015 | 55 | M | Caucasian | GSW - Head | None |
| INBRAIN063 | Depression | 5/15/2015 | 52 | M | Caucasian | Hanging | Alcohol(Ethanol), Stimulants (Caffeine) |
| INBRAIN064 | None | 6/17/2015 | 34 | M | Caucasian | GSW - Head |  |
| INBRAIN065 | ADHD | 11/2/2015 | 19 | F | Caucasian | GSW -Head | Negative |

**Table S3: Co-morbidity.** Clock genes from Table 1. Evidence of involvement in other disorders

| **Gene Symbol/Gene Name** | **(Change in Suicide)**  **Validation p-value INBRAIN**  **Stepwise** | **Full Moon Significant groups Predictions**  **AUC/pvalue** | **Prior human Brain expression evidence for other disorder** | **Prior human peripheral evidence for other disorder** | **Prior Non-human Brain expression evidence for other disorder** | | **Prior Non-human peripheral evidence for other disorder** |
| --- | --- | --- | --- | --- | --- | --- | --- |
| ACSM3 | (D) 9.67E-06 Bonferoni | Full Moon  Age ≤30 yo ACSM3 0.820/0.046 | (D)  superior frontal cortex  **Alcohol**  ^1^  (D)  Male Subic **Depression** ^2^  (D)  Excitatory Neurons  **ALZ no-pathology vs pathology**  ^3^ | (D)  Blood  **Stress**  ^4^  (D)  PBMCs  **Aging**  ^5^  (D) Lymphoblastoid cells  **Alcohol**  ^6^ | (D) CeA  **Alcohol**  {Warden, 2020 #70937} | |  |
| AHCYL2 | (D) 6.28E-05 Bonferoni | Full Moon ALL AHCYL2 0.733/0.016 Full Moon  Age ≥55 yo AHCYL2 0.848/0.037 | (D)  sgACC, OFC Male **MDD**  ^7^  (D)  Brain Female **Depression**  ^8^ |  | (D) AMY (males) **BP** ^9^  (D) AMY (males) **Stress** ^9^  (D) ventral tegmental area (VTA)  **Alcohol**  {Ferguson, 2018 #70938}  (D) nucleus accumbens (NAC) **Alcohol**  ^10^  (D) PFC **Alcohol**  {Smith, 2020 #70939} | |  |
| AK2 | (D) 1.15E-07 Bonferoni | Full Moon  Age ≤30 yo AK2 0.821/0.046 Peak Hour ≥55 AK2 0.955/0.024 | (D)  PFC (BA46)  **BP,SZ**  ^11^  (D) Brain  **Female** **Depression**  ^8^  (D)  Inhibitory Neurons **Cognition, Dementia, ALZ no-pathology vs pathology**  ^3^ | (D) Blood High **Stress** **State**  ^4^  (D)  Blood  **Memory Retention**  ^12^  (D) Blood **Low Mood State**  {Le-Niculescu, 2021 #70933}  (D) PBMCs **PTSD** ^13^  (D) blood **Dementia** ^14^  (D) Lymphoblastoid cell lines **Alcohol**  {McClintick, 2019 #70932}  (D)  SH-SY5Y cells  **Alcohol**  {McClintick, 2020 #70934} | (D) CeA  **Alcohol**  {Warden, 2020 #70937} | |  |
| GSK3B | (D) 2.19E-36 Bonferoni | Peak Hour All GSK3B 0.706/0.05 | (D)  Anterior Cingulate Cortex  **BP**  ^15^  (D)  Brain  **BP**  ^16^  (D)  DLPFC  SZ  ^17^  (D)  DLPFC (BA46)  **BP**  ^18^  (D)  Dorsolateral PFC  **BP**  ^15^  (D)  Hippocampus  **SZ**  ^19^  (D)  Hypothalamus  **Alcohol**  ^20^  (D)  PFC  **Suicide**  ^21^  (D)  PFC  **Suicide**  ^22^  Thalamus  SZ  ^23^  (D)  Locus Coeruleus **Suicide**  ^24^  (D)  Hippocampus **Alcohol**  {Zhou, 2011 #70930}  (D)  Brain  **Depression**  ^8^  (D)  Hippocampus **Alcohol**  {Farris, 2015 #70931}  (D)  astrocytes **Dementia**  ^3^  (D)  Hippocampus  **SZ**  ^25^  (D)  PFC  **SZ**  ^26^  (D)  PFC  **MDD**  ^27^  (D)  PFC  **Suicide**  ^27^ | (D) Blood  **High Pain State** ^28^  (D) Blood  **High Stress State**  ^4^  (D) Blood **Memory Retention**  ^12^  (D) peripheral blood mononuclear cells **Dementia** ^29^  (D) Platelets **BP** ^30^ | (D) Hippocampus **Stress** ^31^  (D) AMY (Males) **Stress** ^32^  (D) DRG **Neuropathic Pain**  {von Schack, 2011 #70940}  (D) PFC **Yohimbine** ^33^  (D) PFC (males) **BP**,,**Stress** ^9^  (D) PFC **Methamphetamine** ^34^ | | (D) Blood (Females) **Stress** ^32^  (D) **Aging** {Dysarz, 2021 #70943}  (D) plasma **Aging** ^35^ |
| PRKCB | (D) 2.40E-13 Bonferoni | Peak Hour ≥55 PRKCB 0.955/0.024 | (D)  Anterior Cingulate Cortex  **BP**  ^36^  (D)  DLPFC  **SZ**  ^37^  (D)  PFC  **Aging**  ^38^  (D) temporal neocoretx **Autism** ^39^  (D) cerebral cortex **Autism** ^40^  (D) Temporal Cortex **Alzheimer's Disease** ^41^  (D) Hippocampus **Alzheimer's Disease** ^42^  (D) Hippocampus **Alzheimer's Disease** ^43^  (D) Dorsolateral Prefrontal Area **Suicide** {Cabrera, 2019 #70921}  (D) Entorhinal cortex **Alzheimer’s Disease** ^44^  (D) associative striatum **SZ** ^45^  (D) dorsolateral prefrontal cortex **Type 2 SZ**  ^37^  (D) Dorsolateral Prefrontal Area **Suicide** {Cabrera, 2019 #70921}  (D) DLPFC **Suicide** ^46^  (D) Cortical tissue **Alzheimer's Disease**{Murano, 2019 #70909}  (D) Prefrontal cortex **ASD** {Murano, 2019 #70909}  (D) Amygdala **Depression** {Murano, 2019 #70909}  (D) putamen **Parkinson** {Murano, 2019 #70909}  (D) prefrontal cortex **Parkinson** {Murano, 2019 #70909}  (D) Brain BA100 **SZ** {Murano, 2019 #70909}  (D) Associative striatum tissues **SZ** {Murano, 2019 #70909}  (D) PFC , hippocampus **Suicide** ^47^  (D) prefrontal cortex **Alzheimer's Disease** ^48^ | (D) Blood **Memory Retention**  ^12^  (D) Blood **Low Mood State** {Le-Niculescu, 2021 #70933}  (D) Peripheral blood monocytes **Chronic Stress** ^49^  (D) Blood **Stress, PTSD**  ^50^  (D) blood **Psychosis** ^51^  (D) Peripheral Blood Leukocyte **SZ** ^52^  (D) iPSCs **ALS** {Murano, 2019 #70909}  (D) embryonic stem cells **ALS** {Murano, 2019 #70909}  (D) Induced pluripotent stem cell line XIST RNA **Huntington's Disease** {Murano, 2019 #70909}  (D) Platelets **BP** ^53^  (D) Platelets **PTSD** ^54^ | (D) Hippocampus anf PFC **Anxiety** ^55^  (D) Female PFC **Stress** ^2^  (D) PFC (paradigm 3) **Alcohol** ^56^  (D) VT **Phencyclidine** ^57^  (D) ventral striatum (VS) **Alcohol** {Ferguson, 2018 #70938}  (D) Central Nucleus of the Amygdala **Alcohol** {Freeman, 2013 #70941} | |  |
| PRKCI | (D) 2.71E-05 Bonferoni | Peak Month  Age≤30 (November) PRKCI 0.893/0.040 | (D) prefrontal cortical parvalbumin neurons cells (PV cells) **SZ** ^58^  (D) Hippocampus **Alzheimer's Disease** ^42^  (D) dorsolateral prefrontal cortex **SZ** ^45^  (D) hippocampus **Aging** ^59^ | (D) Blood **Memory Retention**  ^12^  (D) blood **MDD** ^60^ | (D) PFC **Depression** ^61^  (D) NAC **Stress** ^2^  (D) PFC **Alcohol** {Warden, 2020 #70937}  (D) Central Nucleus of the Amygdala **Alcohol** {Freeman, 2013 #70941} | (D) plasma **Aging** ^35^ | |
| RBM3 | (D) 1.73E-05 Bonferoni | Full Moon  ALL RBM3 0.722/0.021 Full Moon  Age ≥55 yo RBM3 0.939/0.008 Full Moon  Age ≤30 yo RBM3 0.846/0.035 | (D) Cerebellum **MDD** ^62^  (D) Hippocampus **Suicide** ^63^  (D) Temporal lobe **Alzheimer's Disease Progression** ^44^  (D) Entorhinal cortex **Alzheimer's Disease** ^44^  (D) DLPFC Females **Suicide Completers** ^64^  (D) Brain **Depression**  ^8^  (D) Brain Female **Depression**  ^8^  (D) Excitatory Neurons **Alzheimer’s Diseases** ^3^ | (D) Blood **High Stress State** ^4^  (D) Blood **Low Mood State** {Le-Niculescu, 2021 #70933}  (D) leukocytes; blood **Later-Life Depression (LLD)** ^65^  blood **MDD** {Qi, 2021 #70935}  (D) Lymphoblastoid cell lines **Alcohol**  {McClintick, 2019 #70932}  (D) SH-SY5Y cells **Alcohol**  {McClintick, 2020 #70934} | (D) PFC **Stress, MDD** {An, 2020 #70942} | (D) Blood **Methamphetamine** ^66^ | |
| TBL1XR1 | (D) 2.34E-08 Bonferoni | Peak Month  Age≤30 (November) TBL1XR1 0.923/0.028 | (D) Brain **BP** ^16^  (D) cerebral cortex **Alcohol** ^40^  (D) Male BA8/9 **Depression**  ^2^  (D) hippocampus **Aging** ^59^  (D) Anterior Nucleus **SZ** {Bakshi, 2020 #70910}  (D) Brain Male **Depression** ^8^ | (D) Blood **PTSD** ^67^  (D) Blood **PTSD** {Bainomugisa, 2021 #70936} |  | (D) Cell Culture **Alcohol** {Arya, 2013 #70944}  (D) skeletal muscle **Aging** {Kang, 2022 #70945} | |

**Table S4 Biology.** A. Pathway Analyses, B. Diseases of genes in Table 1 (n=8)

| **Pathway Analyses** | | | | | | | | | | | | | | | | |
| --- | --- | --- | --- | --- | --- | --- | --- | --- | --- | --- | --- | --- | --- | --- | --- | --- |
|  | **KEGG Pathways** | | | | | | | **DAVID GO Functional Annotation Biological Processes** | | | | | **Ingenuity Pathways** | | | |
|  | **Term** | | **Count** | **%** | | **P-Value** | | **Term** | | **Count** | **%** | **P-Value** | **Term** | | **Count** | **%** |
|  | Wnt signaling pathway | | 3 | 37.5 | | 6.10E-03 | | cellular response to insulin stimulus | | 3 | 37.5 | 2.50E-03 | ERBB Signaling | | 3.30E-06 | 3.2 % 3/93 |
|  | EGFR tyrosine kinase inhibitor resistance | | 2 | 25 | | 5.70E-02 | | response to insulin | | 3 | 37.5 | 4.50E-03 | Estrogen Receptor Signaling | | 5.97E-06 | 1.0 % 4/409 |
|  | B cell receptor signaling pathway | | 2 | 25 | | 5.90E-02 | | cellular response to peptide hormone stimulus | | 3 | 37.5 | 5.40E-03 | 14-3-3-mediated Signaling | | 8.44E-06 | 2.4 % 3/127 |
|  | ErbB signaling pathway | | 2 | 25 | | 6.10E-02 | | peptidyl-serine phosphorylation | | 3 | 37.5 | 5.50E-03 | GP6 Signaling Pathway | | 8.44E-06 | 2.4 % 3/127 |
|  | Melanogenesis | | 2 | 25 | | 7.20E-02 | | peptidyl-serine modification | | 3 | 37.5 | 6.30E-03 | Factors Promoting Cardiogenesis in Vertebrates | | 1.48E-05 2.0 | 2.0 % 3/153 |
| **Diseases** | | | | | | | | | | | | | | | | |
|  | **David** | | | | | | | | | **Ingenuity Pathways Disease** | | | | | | |
|  | **#** | **Term** | | | **Count** | | **%** | | **P-Value** | **Diseases and Disorders** | | **P-Value** | | **# Molecules** | | |
|  | 1 | plasma HDL cholesterol (HDL-C) levels | | | 2 | | 25 | | 9.30E-02 | Cancer | | 3.23E-02 - 1.01E-07 | | 8 | | |
|  | 2 |  | | |  | |  | |  | Gastrointestinal Disease | | 3.22E-02 - 1.01E-07 | | 6 | | |
|  | 3 |  | | |  | |  | |  | Organismal Injury and Abnormalities | | 3.23E-02 - 1.01E-07 | | 8 | | |
|  | 4 |  | | |  | |  | |  | Dermatological Diseases and Conditions | | 3.12E-02 - 6.67E-06 | | 3 | | |
|  | 5 |  | | |  | |  | |  | Tumor Morphology | | 1.92E-02 - 6.67E-06 | | 3 | | |

**Literature Cited:**

1. Liu J, Lewohl JM, Harris RA, Iyer VR, Dodd PR, Randall PK *et al.* Patterns of gene expression in the frontal cortex discriminate alcoholic from nonalcoholic individuals. *Neuropsychopharmacology* 2006; **31**(7)**:** 1574-1582.

2. Labonte B, Engmann O, Purushothaman I, Menard C, Wang J, Tan C *et al.* Sex-specific transcriptional signatures in human depression. *Nat Med* 2017; **23**(9)**:** 1102-1111.

3. Mathys H, Davila-Velderrain J, Peng Z, Gao F, Mohammadi S, Young JZ *et al.* Single-cell transcriptomic analysis of Alzheimer's disease. *Nature* 2019; **570**(7761)**:** 332-337.

4. Le-Niculescu H, Roseberry K, Levey DF, Rogers J, Kosary K, Prabha S *et al.* Towards precision medicine for stress disorders: diagnostic biomarkers and targeted drugs. *Mol Psychiatry* 2019.

5. Harris SE, Riggio V, Evenden L, Gilchrist T, McCafferty S, Murphy L *et al.* Age-related gene expression changes, and transcriptome wide association study of physical and cognitive aging traits, in the Lothian Birth Cohort 1936. *Aging (Albany NY)* 2017; **9**(12)**:** 2489-2503.

6. Edenberg HJ, Koller DL, Xuei X, Wetherill L, McClintick JN, Almasy L *et al.* Genome-wide association study of alcohol dependence implicates a region on chromosome 11. *Alcohol Clin Exp Res* 2010; **34**(5)**:** 840-852.

7. Girgenti MJ, Wang J, Ji D, Cruz DA, Traumatic Stress Brain Research G, Stein MB *et al.* Transcriptomic organization of the human brain in post-traumatic stress disorder. *Nat Neurosci* 2021; **24**(1)**:** 24-33.

8. Gammie SC. Creation of a gene expression portrait of depression and its application for identifying potential treatments. *Sci Rep* 2021; **11**(1)**:** 3829.

9. Le-Niculescu H, McFarland MJ, Ogden CA, Balaraman Y, Patel S, Tan J *et al.* Phenomic, convergent functional genomic, and biomarker studies in a stress-reactive genetic animal model of bipolar disorder and co-morbid alcoholism. *Am J Med Genet B Neuropsychiatr Genet* 2008; **147B**(2)**:** 134-166.

10. Nagy C, Maitra M, Tanti A, Suderman M, Theroux JF, Davoli MA *et al.* Single-nucleus transcriptomics of the prefrontal cortex in major depressive disorder implicates oligodendrocyte precursor cells and excitatory neurons. *Nat Neurosci* 2020; **23**(6)**:** 771-781.

11. Iwamoto K, Bundo M, Kato T. Altered expression of mitochondria-related genes in postmortem brains of patients with bipolar disorder or schizophrenia, as revealed by large-scale DNA microarray analysis. *Hum Mol Genet* 2005; **14**(2)**:** 241-253.

12. Niculescu AB, Le-Niculescu H, Roseberry K, Wang S, Hart J, Kaur A *et al.* Blood biomarkers for memory: toward early detection of risk for Alzheimer disease, pharmacogenomics, and repurposed drugs. *Mol Psychiatry* 2019.

13. Segman RH, Shefi N, Goltser-Dubner T, Friedman N, Kaminski N, Shalev AY. Peripheral blood mononuclear cell gene expression profiles identify emergent post-traumatic stress disorder among trauma survivors. *Mol Psychiatry* 2005; **10**(5)**:** 500-513, 425.

14. Hogan MM, Vogel SN. Inhibition of macrophage tumoricidal activity by glucocorticoids. *J Immunol* 1988; **140**(2)**:** 513-519.

15. Vawter MP, Tomita H, Meng F, Bolstad B, Li J, Evans S *et al.* Mitochondrial-related gene expression changes are sensitive to agonal-pH state: implications for brain disorders. *Mol Psychiatry* 2006; **11**(7)**:** 615, 663-679.

16. Chen H, Wang N, Zhao X, Ross CA, O'Shea KS, McInnis MG. Gene expression alterations in bipolar disorder postmortem brains. *Bipolar Disord* 2013; **15**(2)**:** 177-187.

17. Kozlovsky N, Shanon-Weickert C, Tomaskovic-Crook E, Kleinman JE, Belmaker RH, Agam G. Reduced GSK-3beta mRNA levels in postmortem dorsolateral prefrontal cortex of schizophrenic patients. *Journal of neural transmission* 2004; **111**(12)**:** 1583-1592.

18. Nakatani N, Hattori E, Ohnishi T, Dean B, Iwayama Y, Matsumoto I *et al.* Genome-wide expression analysis detects eight genes with robust alterations specific to bipolar I disorder: relevance to neuronal network perturbation. *Hum Mol Genet* 2006; **15**(12)**:** 1949-1962.

19. Nadri C, Dean B, Scarr E, Agam G. GSK-3 parameters in postmortem frontal cortex and hippocampus of schizophrenic patients. *Schizophr Res* 2004; **71**(2-3)**:** 377-382.

20. Gonzalez R, Bernardo C, Cruz D, Walss-Bass C, Thompson PM. The relationships between clinical characteristics, alcohol and psychotropic exposure, and circadian gene expression in human postmortem samples of affective disorder and control subjects. *Psychiatry Res* 2014; **218**(3)**:** 359-362.

21. Pandey GN, Dwivedi Y, Rizavi HS, Teppen T, Gaszner GL, Roberts RC *et al.* GSK-3beta gene expression in human postmortem brain: regional distribution, effects of age and suicide. *Neurochem Res* 2009; **34**(2)**:** 274-285.

22. Ren X, Rizavi HS, Khan MA, Dwivedi Y, Pandey GN. Altered Wnt signalling in the teenage suicide brain: focus on glycogen synthase kinase-3beta and beta-catenin. *Int J Neuropsychopharmacol* 2013; **16**(5)**:** 945-955.

23. Chu TT, Liu Y, Kemether E. Thalamic transcriptome screening in three psychiatric states. *J Hum Genet* 2009; **54**(11)**:** 665-675.

24. Roy B, Wang Q, Palkovits M, Faludi G, Dwivedi Y. Altered miRNA expression network in locus coeruleus of depressed suicide subjects. *Sci Rep* 2017; **7**(1)**:** 4387.

25. Torrey EF, Barci BM, Webster MJ, Bartko JJ, Meador-Woodruff JH, Knable MB. Neurochemical markers for schizophrenia, bipolar disorder, and major depression in postmortem brains. *Biol Psychiatry* 2005; **57**(3)**:** 252-260.

26. Blasi G, Napolitano F, Ursini G, Di Giorgio A, Caforio G, Taurisano P *et al.* Association of GSK-3beta genetic variation with GSK-3beta expression, prefrontal cortical thickness, prefrontal physiology, and schizophrenia. *Am J Psychiatry* 2013; **170**(8)**:** 868-876.

27. Karege F, Perroud N, Burkhardt S, Fernandez R, Ballmann E, La Harpe R *et al.* Protein levels of beta-catenin and activation state of glycogen synthase kinase-3beta in major depression. A study with postmortem prefrontal cortex. *J Affect Disord* 2012; **136**(1-2)**:** 185-188.

28. Niculescu AB, Le-Niculescu H, Levey DF, Roseberry K, Soe KC, Rogers J *et al.* Towards precision medicine for pain: diagnostic biomarkers and repurposed drugs. *Mol Psychiatry* 2019; **24**(4)**:** 501-522.

29. Marksteiner J, Humpel C. Glycogen-synthase kinase-3beta is decreased in peripheral blood mononuclear cells of patients with mild cognitive impairment. *Exp Gerontol* 2009; **44**(6-7)**:** 370-371.

30. Pandey GN, Ren X, Rizavi HS, Dwivedi Y. Glycogen synthase kinase-3beta in the platelets of patients with mood disorders: effect of treatment. *J Psychiatr Res* 2010; **44**(3)**:** 143-148.

31. Omata N, Chiu CT, Moya PR, Leng Y, Wang Z, Hunsberger JG *et al.* Lentivirally mediated GSK-3beta silencing in the hippocampal dentate gyrus induces antidepressant-like effects in stressed mice. *Int J Neuropsychopharmacol* 2011; **14**(5)**:** 711-717.

32. Daskalakis NP, Cohen H, Cai G, Buxbaum JD, Yehuda R. Expression profiling associates blood and brain glucocorticoid receptor signaling with trauma-related individual differences in both sexes. *Proc Natl Acad Sci U S A* 2014; **111**(37)**:** 13529-13534.

33. Le-Niculescu H, Balaraman Y, Patel SD, Ayalew M, Gupta J, Kuczenski R *et al.* Convergent functional genomics of anxiety disorders: translational identification of genes, biomarkers, pathways and mechanisms. *Transl Psychiatry* 2011; **1:** e9.

34. Ogden CA, Rich ME, Schork NJ, Paulus MP, Geyer MA, Lohr JB *et al.* Candidate genes, pathways and mechanisms for bipolar (manic-depressive) and related disorders: an expanded convergent functional genomics approach. *Mol Psychiatry* 2004; **9**(11)**:** 1007-1029.

35. Lehallier B, Gate D, Schaum N, Nanasi T, Lee SE, Yousef H *et al.* Undulating changes in human plasma proteome profiles across the lifespan. *Nat Med* 2019; **25**(12)**:** 1843-1850.

36. Tomita H, Ziegler ME, Kim HB, Evans SJ, Choudary PV, Li JZ *et al.* G protein-linked signaling pathways in bipolar and major depressive disorders. *Frontiers in genetics* 2013; **4:** 297.

37. Bowen EFW, Burgess JL, Granger R, Kleinman JE, Rhodes CH. DLPFC transcriptome defines two molecular subtypes of schizophrenia. *Transl Psychiatry* 2019; **9**(1)**:** 147.

38. Erraji-Benchekroun L, Underwood MD, Arango V, Galfalvy H, Pavlidis P, Smyrniotopoulos P *et al.* Molecular aging in human prefrontal cortex is selective and continuous throughout adult life. *Biol Psychiatry* 2005; **57**(5)**:** 549-558.

39. Lintas C, Sacco R, Garbett K, Mirnics K, Militerni R, Bravaccio C *et al.* Involvement of the PRKCB1 gene in autistic disorder: significant genetic association and reduced neocortical gene expression. *Mol Psychiatry* 2009; **14**(7)**:** 705-718.

40. Gandal MJ, Haney JR, Parikshak NN, Leppa V, Ramaswami G, Hartl C *et al.* Shared molecular neuropathology across major psychiatric disorders parallels polygenic overlap. *Science* 2018; **359**(6376)**:** 693-697.

41. Castillo E, Leon J, Mazzei G, Abolhassani N, Haruyama N, Saito T *et al.* Comparative profiling of cortical gene expression in Alzheimer's disease patients and mouse models demonstrates a link between amyloidosis and neuroinflammation. *Sci Rep* 2017; **7**(1)**:** 17762.

42. Blalock EM, Geddes JW, Chen KC, Porter NM, Markesbery WR, Landfield PW. Incipient Alzheimer's disease: microarray correlation analyses reveal major transcriptional and tumor suppressor responses. *Proc Natl Acad Sci U S A* 2004; **101**(7)**:** 2173-2178.

43. van Rooij JGJ, Meeter LHH, Melhem S, Nijholt DAT, Wong TH, Netherlands Brain B *et al.* Hippocampal transcriptome profiling combined with protein-protein interaction analysis elucidates Alzheimer's disease pathways and genes. *Neurobiol Aging* 2019; **74:** 225-233.

44. Patel H, Hodges AK, Curtis C, Lee SH, Troakes C, Dobson RJB *et al.* Transcriptomic analysis of probable asymptomatic and symptomatic alzheimer brains. *Brain Behav Immun* 2019; **80:** 644-656.

45. Lanz TA, Reinhart V, Sheehan MJ, Rizzo SJS, Bove SE, James LC *et al.* Postmortem transcriptional profiling reveals widespread increase in inflammation in schizophrenia: a comparison of prefrontal cortex, striatum, and hippocampus among matched tetrads of controls with subjects diagnosed with schizophrenia, bipolar or major depressive disorder. *Transl Psychiatry* 2019; **9**(1)**:** 151.

46. Cabrera-Mendoza B, Fresno C, Monroy-Jaramillo N, Fries GR, Walss-Bass C, Glahn DC *et al.* Brain Gene Expression Profiling of Individuals With Dual Diagnosis Who Died by Suicide. *J Dual Diagn* 2019**:** 1-14.

47. Pandey GN, Dwivedi Y, Rizavi HS, Ren X, Conley RR. Decreased catalytic activity and expression of protein kinase C isozymes in teenage suicide victims: a postmortem brain study. *Arch Gen Psychiatry* 2004; **61**(7)**:** 685-693.

48. Garranzo-Asensio M, San Segundo-Acosta P, Martinez-Useros J, Montero-Calle A, Fernandez-Acenero MJ, Haggmark-Manberg A *et al.* Identification of prefrontal cortex protein alterations in Alzheimer's disease. *Oncotarget* 2018; **9**(13)**:** 10847-10867.

49. Miller GE, Chen E, Sze J, Marin T, Arevalo JM, Doll R *et al.* A functional genomic fingerprint of chronic stress in humans: blunted glucocorticoid and increased NF-kappaB signaling. *Biol Psychiatry* 2008; **64**(4)**:** 266-272.

50. Mehta D, Klengel T, Conneely KN, Smith AK, Altmann A, Pace TW *et al.* Childhood maltreatment is associated with distinct genomic and epigenetic profiles in posttraumatic stress disorder. *Proc Natl Acad Sci U S A* 2013; **110**(20)**:** 8302-8307.

51. Leirer DJ, Iyegbe CO, Di Forti M, Patel H, Carra E, Fraietta S *et al.* Differential gene expression analysis in blood of first episode psychosis patients. *Schizophr Res* 2019; **209:** 88-97.

52. Zhang Y, You X, Li S, Long Q, Zhu Y, Teng Z *et al.* Peripheral Blood Leukocyte RNA-Seq Identifies a Set of Genes Related to Abnormal Psychomotor Behavior Characteristics in Patients with Schizophrenia. *Med Sci Monit* 2020; **26:** e922426.

53. Pandey GN, Ren X, Dwivedi Y, Pavuluri MN. Decreased protein kinase C (PKC) in platelets of pediatric bipolar patients: effect of treatment with mood stabilizing drugs. *J Psychiatr Res* 2008; **42**(2)**:** 106-116.

54. Koenigsberg HW, Yuan P, Diaz GA, Guerreri S, Dorantes C, Mayson S *et al.* Platelet protein kinase C and brain-derived neurotrophic factor levels in borderline personality disorder patients. *Psychiatry Res* 2012; **199**(2)**:** 92-97.

55. Virok DP, Kis Z, Szegedi V, Juhasz G, Zvara A, Jr., Muller G *et al.* Functional changes in transcriptomes of the prefrontal cortex and hippocampus in a mouse model of anxiety. *Pharmacol Rep* 2011; **63**(2)**:** 348-361.

56. Rodd ZA, Bertsch BA, Strother WN, Le-Niculescu H, Balaraman Y, Hayden E *et al.* Candidate genes, pathways and mechanisms for alcoholism: an expanded convergent functional genomics approach. *Pharmacogenomics J* 2007; **7**(4)**:** 222-256.

57. Le-Niculescu H, Balaraman Y, Patel S, Tan J, Sidhu K, Jerome RE *et al.* Towards understanding the schizophrenia code: an expanded convergent functional genomics approach. *Am J Med Genet B Neuropsychiatr Genet* 2007; **144B**(2)**:** 129-158.

58. Enwright Iii JF, Huo Z, Arion D, Corradi JP, Tseng G, Lewis DA. Transcriptome alterations of prefrontal cortical parvalbumin neurons in schizophrenia. *Mol Psychiatry* 2018; **23**(7)**:** 1606-1613.

59. Jaffe AE, Hoeppner DJ, Saito T, Blanpain L, Ukaigwe J, Burke EE *et al.* Profiling gene expression in the human dentate gyrus granule cell layer reveals insights into schizophrenia and its genetic risk. *Nat Neurosci* 2020; **23**(4)**:** 510-519.

60. de Kluiver H, Jansen R, Milaneschi Y, Penninx B. Involvement of inflammatory gene expression pathways in depressed patients with hyperphagia. *Transl Psychiatry* 2019; **9**(1)**:** 193.

61. Malki K, Pain O, Tosto MG, Du Rietz E, Carboni L, Schalkwyk LC. Identification of genes and gene pathways associated with major depressive disorder by integrative brain analysis of rat and human prefrontal cortex transcriptomes. *Transl Psychiatry* 2015; **5:** e519.

62. Forero DA, Guio-Vega GP, Gonzalez-Giraldo Y. A comprehensive regional analysis of genome-wide expression profiles for major depressive disorder. *J Affect Disord* 2017; **218:** 86-92.

63. Labonte B, Suderman M, Maussion G, Lopez JP, Navarro-Sanchez L, Yerko V *et al.* Genome-wide methylation changes in the brains of suicide completers. *Am J Psychiatry* 2013; **170**(5)**:** 511-520.

64. Cabrera-Mendoza B, Fresno C, Monroy-Jaramillo N, Fries GR, Walss-Bass C, Glahn DC *et al.* Sex differences in brain gene expression among suicide completers. *J Affect Disord* 2020; **267:** 67-77.

65. Miyata S, Yamagata H, Matsuo K, Uchida S, Harada K, Fujihara K *et al.* Characterization of the signature of peripheral innate immunity in women with later-life major depressive disorder. *Brain Behav Immun* 2020; **87:** 831-839.

66. Le-Niculescu H, Kurian SM, Yehyawi N, Dike C, Patel SD, Edenberg HJ *et al.* Identifying blood biomarkers for mood disorders using convergent functional genomics. *Mol Psychiatry* 2009; **14**(2)**:** 156-174.

67. Breen MS, Maihofer AX, Glatt SJ, Tylee DS, Chandler SD, Tsuang MT *et al.* Gene networks specific for innate immunity define post-traumatic stress disorder. *Mol Psychiatry* 2015; **20**(12)**:** 1538-1545.
